# Analysis of crystal structure of *Arabidopsis* MPK6 and generation of its mutants with higher activity

**DOI:** 10.1038/srep25646

**Published:** 2016-05-10

**Authors:** Bo Wang, Xinghua Qin, Juan Wu, Hongying Deng, Yuan Li, Hailian Yang, Zhongzhou Chen, Guoqin Liu, Dongtao Ren

**Affiliations:** 1 State Key Laboratory of Plant Physiology and Biochemistry, College of Biological Sciences, China Agricultural University, Beijing 100193, China

## Abstract

Mitogen-activated protein kinase (MAPK) cascades, which are the highly conserved signalling modules in eukaryotic organisms, have been shown to play important roles in regulating growth, development, and stress responses. The structures of various MAPKs from yeast and animal have been solved, and structure-based mutants were generated for their function analyses, however, the structures of plant MAPKs remain unsolved. Here, we report the crystal structure of *Arabidopsis* MPK6 at a 3.0 Å resolution. Although MPK6 is topologically similar to ERK2 and p38, the structures of the glycine-rich loop, MAPK insert, substrate binding sites, and L16 loop in MPK6 show notable differences from those of ERK2 and p38. Based on the structural comparison, we constructed MPK6 mutants and analyzed their kinase activity both *in vitro* and *in planta*. MPK6^F364L^ and MPK6^F368L^ mutants, in which Phe364 and Phe368 in the L16 loop were changed to Leu, respectively, acquired higher intrinsic kinase activity and retained the normal MAPKK activation property. The expression of MPK6 mutants with basal activity is sufficient to induce camalexin biosynthesis; however, to induce ethylene and leaf senescence, the expression of MPK6 mutants with higher activity is required. The results suggest that these mutants can be used to analyze the specific biological functions of MPK6.

Mitogen-activated protein kinase (MAPK) cascades, which are composed of MAPK kinase kinase, MAPK kinase and MAPK (called MAPKKK, MAPKK, and MAPK), are highly conserved signalling modules in eukaryotic organisms. MAPKKKs can be activated by other kinases or sensors/receptors. MAPKKs are activated by MAPKKK via the phosphorylation of the two serine and threonine residues in their conserved S/T-X_3**–**5_-S/T motif. MAPKKs are dual-specificity kinases that activate MAPKs by phosphorylating the threonine and tyrosine residues in their conserved TXY motif. The activated MAPKs have been shown to regulate specific cellular processes primarily through the phosphorylation of different substrates; however, several MAPKs (such as ERK1 and ERK2 in animals) were also shown to perform their function only by binding to targets in an kinase activity-independent manner^1^.

Crystallography and mutagenesis analyses of MAPKs and MAPK-substrate complexes have revealed the mechanism by which MAPKs and their substrates interact. The structures of mammalian and yeast MAPKs have been studied^2^. Several structural elements within MAPKs that participated in the regulation of kinase activity and interaction with their substrates were defined. The overall structures of the MAPKs, which have been reported, possess an N-terminal lobe and a C-terminal lobe. A deep cleft between the N- and C-lobes forms the ATP-binding site. With the exception of the common structural elements found in other kinases, MAPKs also contain a few unique features, including a MAPK insert and a C-terminal αL16 helix^3^,^4^,^5^,^6^.

Since the first reports of plant MAPKs, alfalfa MsERK1 and pea D5 kinase in 1993^7^,^8^, a great number of MAPKKKs, MAPKKs, and MAPKs have been identified in plants^9^,^10^,^11^. Over the past two decades, the MAPK cascades in plants have been shown to be important signalling modules that coordinate stress and hormone responses, innate immunity, and plant growth and development^11^,^12^,^13^. The full family members of the MAPK cascades have been defined in several plant species in which the genome sequences have been determined^9^,^14^,^15^. The *Arabidopsis* genome contains 60 MKKKs, 10 MKKs and 23 MPKs (according to systemic nomenclature, the MAPKKKs, MAPKKs and MAPKs in *Arabidopsis* were called MKKKs, MKKs and MPKs)^9^. The MPKs were classified into four groups: the A to C groups contain the conserved TEY motif, and the D group contains the TDY motif^9^. To date, *Arabidopsis* MPK3 and MPK6 in group A and MPK4 in group B are the plant MAPKs that have undergone the most functional analyses. *Arabidopsis* MPK3 and MPK6 were shown to be activated by multiple MKKs, including MKK2, MKK3, MKK4, MKK5, MKK7, and MKK9, and are involved in the regulation of biotic and abiotic stress responses and growth and development processes^16^,^17^,^18^,^19^,^20^,^21^,^22^,^23^,^24^,^25^. Activated MPK6 phosphorylates a variety of substrates, including enzymes, transcription factors, and cellular structural proteins^21^,^26^,^27^,^28^,^29^,^30^,^31^. The complexity of the upstream MKKs and downstream substrates suggests that plant MPKs should possess specific mechanisms to accurately interact with these MKKs and substrates. Crystal structures are useful for understanding these mechanisms; however, no crystal structure of a plant MPK has yet been reported.

Here, we present the crystal structure of the unphosphorylated *Arabidopsis* MPK6 at 3.0 Å resolution. The structure reveals that although MPK6 is topologically similar to the non-plant MAPKs, the structures of the glycine-rich loop, MAPK insert, and L16 loop in MPK6 are saliently different from those of MAPKs. Based on the structural information, various MPK6 mutants were generated. Using recombinant proteins and *Arabidopsis* transgenic plants, we analyzed the kinase activity of the MPK6 mutants and their biological function. We showed that the expression of MPK6 mutants with basal activity is sufficient to induce camalexin biosynthesis. However, to induce ethylene and leaf senescence, the expression of MPK6 mutants with higher activity is required.

## Results

### Crystallization of *Arabidopsis* MPK6

**Figure 1:**
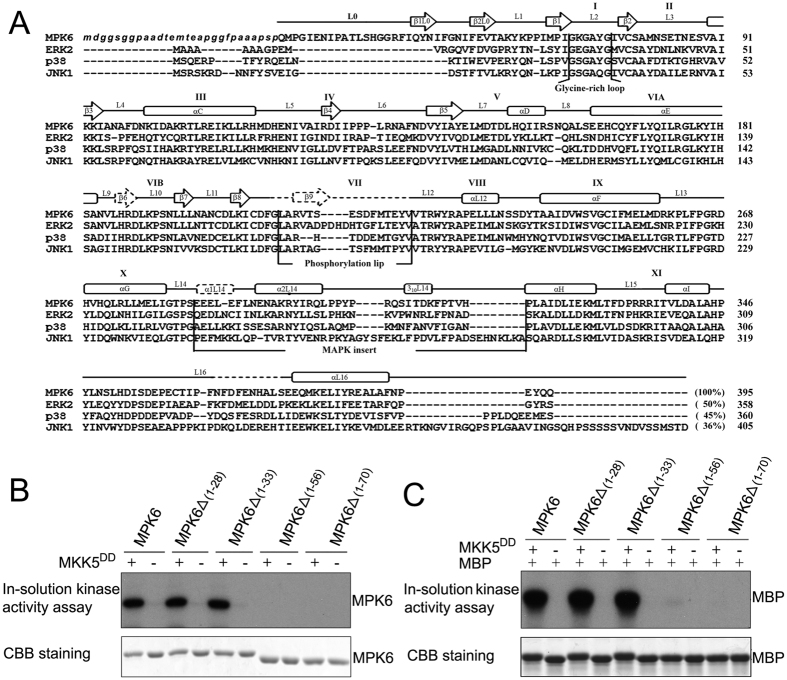
Structure-based sequence alignment of *Arabidopsis* MPK6 with several non-plant MAPKs and kinase activity assays of the different truncated MPK6 proteins. (**A**) Multiple sequence alignment of *Arabidopsis* MPK6 (PDB code: 5CI6), rat ERK2 (PDB code: 1ERK), mouse p38 (PDB code: 1P38), and human JNK1 (PDB code: 1UKH). MPK6 shares 50% sequence identity with ERK2, 45% with p38, and 36%with JNK1. The roman numerals indicate the subdomains of the MAPKs. The residues written in lowercase and italicized letters indicate the residues that were not included in the structure but were used for the crystallographic studies. The secondary structure elements of MPK6 are indicated above the sequences, with open boxes representing the α and 3/10 helices and open arrows representing the β strands. The dashed lines denote the secondary structures that were presented in ERK2 but not MPK6. The glycine-rich loop, phosphorylation lip, and MAPK insert are also labelled. (**B**) *In vitro* phosphorylation of the recombinant MPK6 protein and its truncated mutant proteins by the active MKK5 (MKK5^DD^). (**C**) Kinase activity of the recombinant MPK6 protein and its truncated mutants after phosphorylation by the active MKK5 (MKK5^DD^). Myelin basic protein was used as the substrate.

Initially, the full-length MPK6 protein was used to grow crystals in this study. However, no crystals were obtained after testing various crystallization conditions. The protein sequence alignment shows that MPK6 has an extended N terminus compared with a variety of mammalian MAPKs whose structures have been reported (Fig. 1). Because this extended region of MPK6 is disordered in the predicted secondary structure, we reasoned that the N terminus of MPK6 may be flexible and might interfere with crystal formation. It has also been reported that the flexible N- and C-termini interfered with the crystal formation of JNK1 and JNK3α1^5^,^32^. Thus, truncated MPK6 proteins with the different N-terminal deletions, which can be activated by the upstream MKK, were screened and used for further crystallization. Upon phosphorylation by upstream MKKs, MPK6 was previously shown to be activated and could phosphorylate its substrates *in vitro* and *in planta*^17^,^18^,^19^,^22^,^23^,^24^,^28^,^30^. The bacterially expressed MPK6 and its truncated forms were purified, and their kinase activities were analyzed using myelin basic protein (MBP) as substrate. Consistent with the previous reports^19^,^22^, MPK6 could be phosphorylated and activated by MKK5^DD^, which is an active form of MKK5 (Fig. 1B,C). MPK6_Δ1–56_ and MPK6_Δ1–70_, mutants with 1–56 and 1–70 amino acid residue deletions respectively, could not be phosphorylated and activated by MKK5^DD^. MPK6_Δ1–28_, a mutant with the shortest amino acid residue deletion in our experiment (with 1–28 amino acid residues deleted), was shown to be phosphorylated and activated by MKK5^DD^ to a similar level as MPK6. The result suggests that MPK6_Δ1–28_ retains the full kinase activity of MPK6. This form of the protein was crystallized using the sitting-drop vapour-diffusion method, and crystals were obtained.

### Overall Structure of MPK6

The crystal structure of MPK6 was determined using the diffraction data and refined to 3.0 Å resolution with a *R*_*work*_/*R*_*free*_ of 22.6/27.2. The statistics on data processing and structural refinement are listed in Table 1. The present model of MPK6 includes amino acid residues 29 to 395.

**Figure 2:**
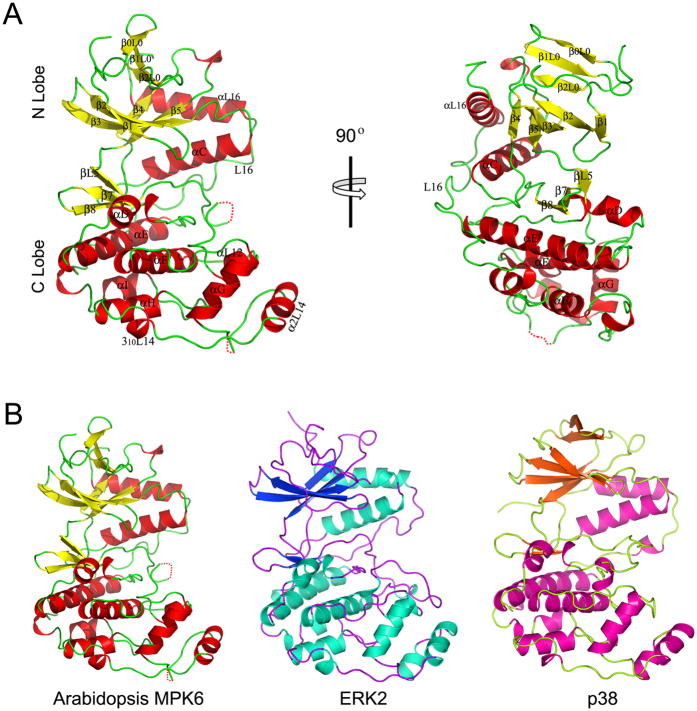
Overall structure of MPK6 and comparison with ERK2 and p38. (**A**) Ribbon representation of the overall MPK6 structure. Colour coding: red, α helices; yellow, β-strands; green, loops. The missing part of the MKI is shown by the red dotted lines. A 90°-rotated view is shown on the right. (**B**) Comparison of the MPK6, ERK2, and p38 structures. All of the structure figures were created in PyMOL.

The MPK6 crystals contain two protein chains in the asymmetric unit. Amino acid residues 70–75 (part of the glycine-rich loop), 213–214 (part of the phosphorylation lip), and 366–374 (part of the L16 loop) in chain A and amino acid residues 215–224 (phosphorylation lip) and 285–287 (part of the MAPK insert) in chain B were not resolved in the electron density maps. The overall structure of MPK6 contains a two-domain structure that is common to protein kinases (Fig. 2)^33^. The N-lobe (residues 32–147 and 360–394) contains eight β-strands (β1 to β5, β0L0, β1L0, and β2L0) and two α-helices (αC and αL16), whereas the C-lobe (residues 149–359) consists of six α-helices (αD to αI) and three short β-strands (β6-β8). The N- and C-lobes are linked by L7 (residues 145–147) and L16 (residues 345–375). The deep cleft between the lobes is the ATP binding pocket. MPK6 also contains the common structural features found in all resolved MAPK structures, including an N-terminal β-hairpin (β1-β5), MAPK insert, and C-terminal αL16 helix.

Compared with other solved protein kinase structures, the overall topology of MPK6 is mostly similar to two well-studied mammalian MAPKs, ERK2 (PDB Code: 1ERK; from rat) and p38 (PDB Code: 1P38; from mouse), with which it shares 50% and 45% amino acid sequence identity, respectively (Fig. 1). The root mean square (rms) deviations are 1.049 Å for ERK2 and 2.330 Å for p38. Independent superimposition of the N- and C-lobes of MPK6 onto ERK2 revealed rms deviations of 1.521 Å for the N-lobe and 0.654 Å for the C-lobe. The rms deviation for the C-lobe was reduced to 0.708 Å when the phosphorylation lip region was ignored. An independent superimposition of the N- and C-lobes of MPK6 onto p38 revealed rms deviations of 1.535 Å for the N-lobe and 0.864 Å for the C-lobe. Because the C- and N-lobes of MPK6 superimpose well with the lobes of these MAPKs, we can map the important residues in MPK6 using their analogous residues in these MAPKs. The major conformational differences between MPK6 and these MAPKs are observed in the ATP binding pocket, the MAP kinase insert, and the L16 loop.

### ATP binding pocket of MPK6

In MPK6, β1 (residues 67 to 70), the glycine-rich loop (residues 71 to 75), and β2 (residues 76 to 82) form a β-L-β structure. The glycine-rich loop in the β-L-β structure, together with αC, αD, L5, L7, β7, β8, DFG motif in L12, and Lys92 in β3, comprise an ATP binding pocket (Fig. 2A). In addition, αE stabilizes the ATP binding pocket through its interactions with β7and β8.

**Figure 3:**
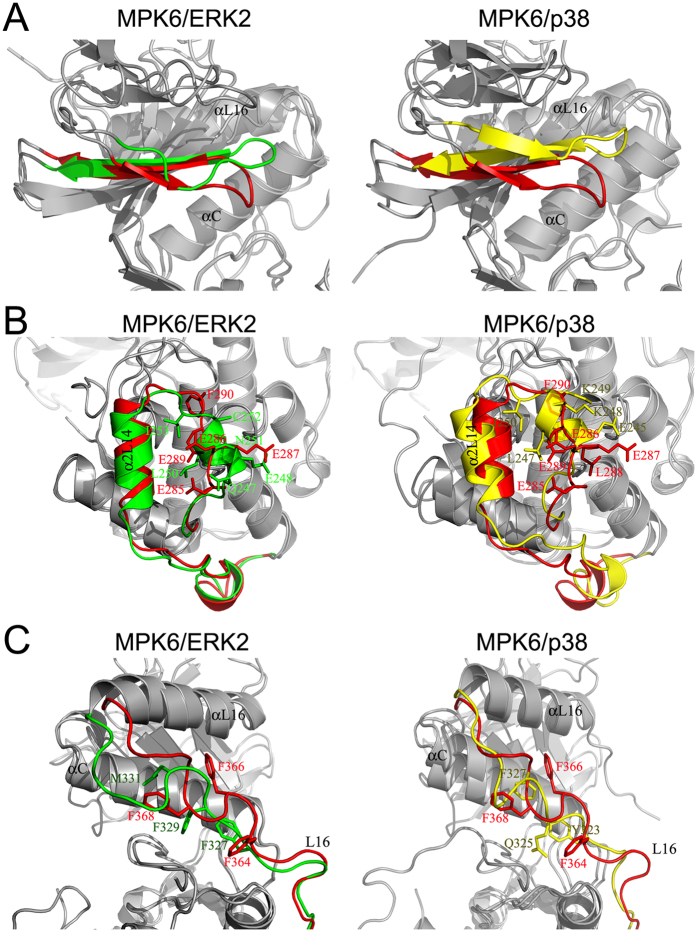
Comparison of the glycine-rich loop, MKI, and L16 loop in MPK6 (red) with those in ERK2 (green) and p38 (yellow). (**A**) The local superposition of the glycine-rich loop in MPK6 with that in ERK2 and p38 showed that the glycine-rich loop in MPK6 was bent towards the ATP-binding pocket. (**B**) The local superposition of the MKI in MPK6 with that in ERK2 and p38 showed the dramatic conformational changes in the α1L14 position of MPK6. (**C**) The local superposition of the L16 loop in MPK6 with that in ERK2 and p38 showed large conformational changes between the structures.

By superimposing the ATP binding pocket in MPK6 with that in ERK2, the MPK6 residues that might participate in ATP binding can be identified (Figs 2 and 3A). Asn145 (Asp104) (the numbers in parentheses refer to the analogous position in ERK2) and Met147 (Met106) in L7 interact with the adenine ring of ATP, and Asp150 (Asp109) in L7 and Gln153 (Lys112) in αD interact with the ATP ribose ring. Asp208 (Asp165) in the DFG motif interacts with Mg^2+^, which, in turn, binds phosphates in ATP.

The glycine-rich loop, which contains a GXGXXGXX consensus sequence, has been reported to play important role in binding and orienting ATP or its derivatives in many protein kinases^34^. The glycine-rich loop in MPK6 showed few changes compared with ERK2 and p38 (Fig. 3A). First, Ala73 and Tyr74 in the loop of MPK6 bend approximately 6.7 Å and 5.4 Å closer towards the ATP binding cleft than do Ala33 and Tyr34 in the loop of inactive ERK2, respectively. Second, the distance between the loop and the phosphorylation lip of MPK6 (residue Asp207 of αC) is 9.6 Å compared with 14.1 Å in ERK2 and 12.7 Å in p38. The differences could be caused by the different interactions between the residues in the glycine-rich loop and residues in the surrounding structures in these MAPKs. Further analysis showed that hydrogen bonds were not formed between the residues in the loop of MPK6 and the residues in the surrounding structures; however, hydrogen bonds were formed by Gly14 and Arg13 in β2L0 with Glu31 and Ala33 in the loop of ERK2 (PDB code: 1ERK), and Ser56 in L4 with Gly34 and Tyr35 in the loop of p38 (PDB code: 1P38).

### MAP kinase insert of MPK6

The MAP kinase insert (MKI), an insertion between helices G and H in the C-lobe of the kinase structure, is a unique structural element that is found in MAPKs and cyclin-depended kinase 2 (CDK2)-related kinases, but not in other protein kinases^3^,^35^,^36^. The MKI in most MAPKs, whose structures have been solved contain α1L14 and α2L14 helices and a 3/10 helix. The MKI was shown to be important for the regulation of ERK2 localization and interaction with upstream MEK1/2 and other binding proteins^37^ and for the binding of p38α to lipids^35^. The MKI of MPK6 is composed of residues 285 to 318 in the C-lobe. In chain A, the MKI of MPK6 is not fully defined due to the absence of electron density for residues 285–287; however, in chain B, the MKI is well resolved.

The MKI of MPK6 undergoes large conformation changes compared with other non-plant MAPKs (Fig. 3B). First, the MKI of MPK6 does not form α1L14 (285–289) but only contains α2L14 (residues 293–301) and a 3/10 helix. Sequence alignment showed that α1L14 in ERK2 (residues 247–252), p38 (residues 244–249) and JNK1 (246–251) contains six amino acid residues, but that the analogous region in MPK6 contains five amino acid residues (residues 285–289), four of which are Glu residues (Glu285-Glu287 and Glu289 in Fig. 1). Superposition of the MKI of MPK6 onto the MKIs of ERK2 and p38 showed that Glu285, Glu286 and Glu287 were not arranged similarly to their analogous residues (Fig. 3B). The Glu cluster created an acidic patch that might interfere with the formation of α1L14 in MPK6. This conclusion was supported by the result showing that the hydrogen bond required for the formation of α1L14 was observed between Gln247 and Leu250 of ERK2 (PDB code: 1ERK) but not between Glu 285 and Leu288 in MPK6. Second, the hydrogen bond formation in the MKI of MPK6 is saliently different from that observed in other non-plant MAPKs. For the MKI of ERK2, hydrogen bonds were formed between Asp249 in α1L14 and Asn295 in L15, between Tyr261 in α2L14 and Asn236 in αG, and between Arg259 in α2L14 with Leu250 and Ile253 in α1L14 (PDB code: 1ERK). The hydrogen bonds between Trp197 in L12 and Ser251 in the linker between α1L14 and α2L14, between Glu245 in α1L14 and Lys295 in L15, and between Lys249 in α1L14 with Asp292 and Asp294 were created in the MKI of p38α (PDB code: 1P38). However, no hydrogen bonds were observed between the residues in MKI and the residues in the surrounding structure of MPK6.

### Substrate binding sites of MPK6

The identified substrate binding sites in the solved MAPK structures mainly lie in the C-terminal domain. Interactions between the D-motif binding site in MAPKs and the docking motifs in substrates, MAPKKs and phosphatases were known to determinate the specificity of recognition. The D-motif binding site consists of the common docking domain (also known as the CD-domain) and hydrophobic docking groove^38^. The hydrophobic docking groove in MAPKs spans across αD to αE in a crevice formed between the helices and β7-β8 reverse turn. The hydrophobic docking groove in MPK6 is significantly different from that in ERK2 and p38. Thr149 blocks the site occupied by φB in p38, similar to Thr108 in ERK2, and there is His165 but not arginine in the φA-2 position. Therefore it will not block that position, similar to JNK1. However, Q159 in MPK6 protrudes into the hydrophobic groove more than Q117 does in ERK2; therefore, the hydrophobic docking groove of MPK6 is much narrower.

### L16 loop of MPK6

With the exception of the MKI, the extension in the C-terminus that spans from the C- to N-lobe is another signature of MAPK that is distinct from other protein kinases^3^,^5^,^39^,^40^. This extension, known as the L16 loop, consists of L16 and aL16. The conformational changes in the L16 loop have been suggested to play an important role in maintaining MAPK basal activity and in MAPK activation^40^,^41^,^42^. L16 of ERK2 (308–338 ) and p38 (305–334) occupy a surface groove formed at the interface between αC and αE and near the phosphorylation lip^3^. Phe329 in L16 of ERK2 is buried against αC; however, upon activation, a new 3/10 helix (residues Phe327 to Leu333) in L16 of ERK2-P2 is formed and leads to Phe329 exposure on the surface and interacts with the lip^40^. In inactive p38, residues Gln325 and Arg330 form a 3/10 helix, but the helix is formed between residues Asp324 to Glu328 and Ser326 to Ser329, respectively, in active p38 (p38^D176A/F327L^ and p38^D176A/F327S^ mutants, and dual phosphorylation activated p38)^41^,^42^,^43^. The structure-based sequence alignment of MPK6 with the other non-plant MAPKs showed that the region spanning residues 345 to 375 comprise the L16 loop of MPK6 (Figs 1A and 2). Compared to ERK2 and p38, the structure of the MPK6 L16 loop does not fit in the groove, due to hindrance from the side chains of Phe364, Phe366, and Phe368 in L16. The L16 conformation induces an 11.4° rotation of αL16 in MPK6 compared with ERK2. Additionally, the 3/10 helix in L16 of inactive p38 does not form in MPK6.

### Kinase activities of MPK6 and its mutants

Although the structural analysis revealed that MPK6 contains structural features that are common to MAPKs of other species, several marked variations were also observed in the MPK6 structure. Previous studies showed that mutations in the ATP binding site of non-plant MAPKs resulted in kinase inactivation, whereas mutations in the L16 loop and MKI of the MAPKs led to kinase autoactivation. To understand the function of these variations, we generated various MPK6 mutants. MPK6^K92R^ is a mutant in which Lys92 in the putative ATP binding site was changed to Arg (R). MPK6^F364L^, MPK6^F366L^, and MPK6^F368L^ are mutants in which Phe364, Phe366, and Phe368 in the L16 loop of MPK6 were changed to Leu. MPK6^E286S^ and MPK6^E289G^ are mutants in which Glu286 and Glu289 in the MKI of MPK6 were changed to Ser and Gly, respectively. The kinase activities of MPK6 and the mutants were assayed.

**Figure 4:**
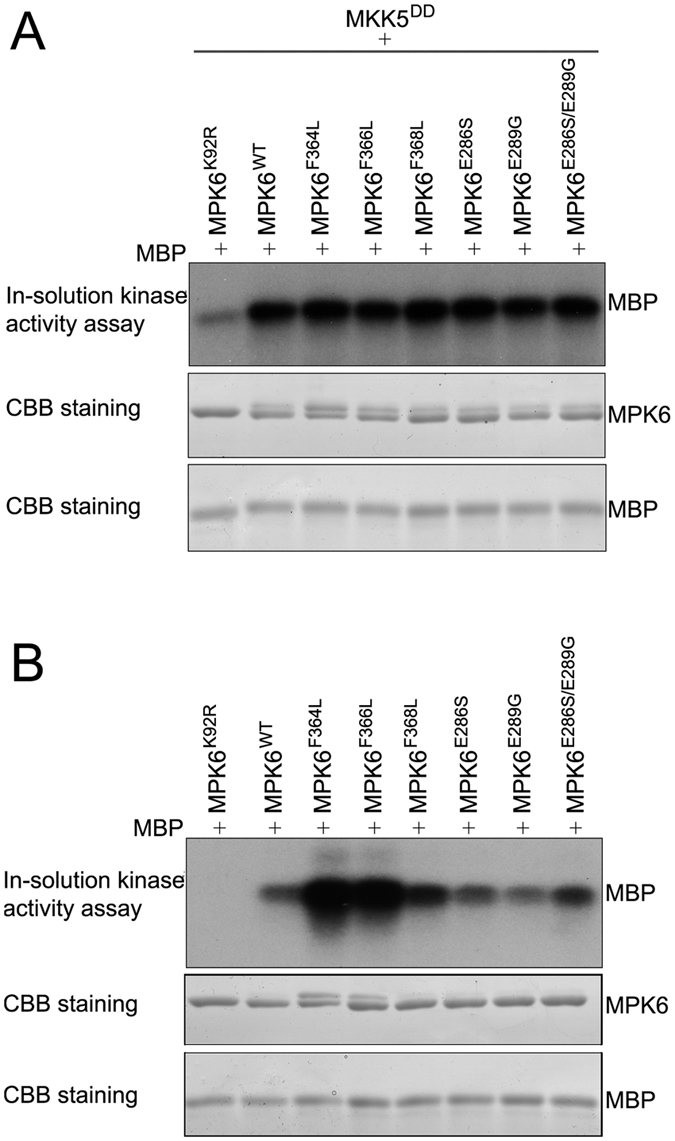
*In vitro* kinase activity assays of MPK6 and its mutants with or without the active MKK5 (MKK5^DD^). The recombinant proteins for the active MKK5 and MPK6 mutants were expressed in *E. coli*, and purified, and subjected to an in-solution kinase assay using myelin basic protein as the substrate. MBP phosphorylation was shown in top panel. The coomassie bright blue (CBB)-stained gels showed that equal amounts of the MPK6 mutants (middle panel) and MBP (bottom panel) were used in each reaction. (**A**) Phosphorylation of the MBP substrate by the MPK6 mutants in the presence of active MKK5 (MKK5^DD^). (**B**) Phosphorylation of the MBP substrate by the MPK6 mutants in the absence of active MKK5 (MKK5^DD^).

To test the kinase activities of MPK6 and its mutants *in vitro*, the recombinant proteins were expressed in *E. coli* and purified. In the presence of active MKK5 (MKK5^DD^), the purified recombinant MPK6 and its mutants, except MPK6^K92R^, showed almost equally high levels of kinase activity using MBP as a substrate (Fig. 4A). However, recombinant MPK6 and its mutants showed different levels of kinase activity in the absence of active MKK5 (MKK5^DD^) (Fig. 4B). MPK6, MPK6^E286S^, and MPK6^E286S/E289G^ exhibited a basal level of kinase activity, whereas MPK6^F364L^, MPK6^F366L^, and MPK6^F368L^ showed significantly higher levels of kinase activity and MPK6^E289G^ showed slightly reduced level of kinase activity compared with MPK6. The results suggest that MPK6 and its mutants, except for MPK6^K92R^, can be fully activated by upstream MAPKK (here as MKK5), and the conformation changes in the L16 loop and MKI alter the kinase activity of MPK6.

**Figure 5:**
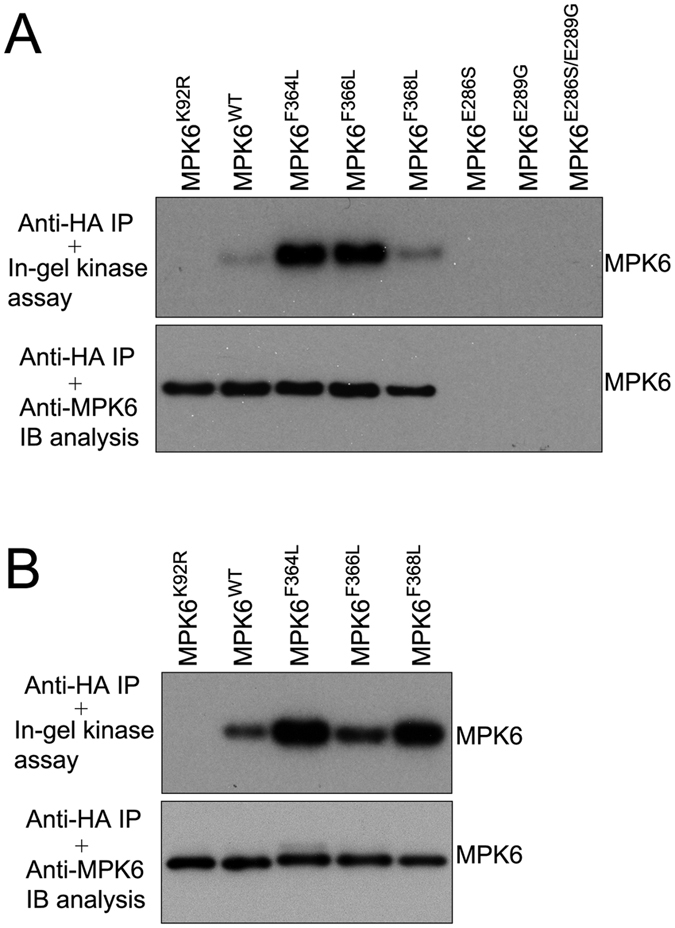
*In planta* kinase activity assays of the MPK6 mutants. The MPK6 mutant proteins were immuno-precipitated (IP) from plants and subjected to an in-gel kinase assay with MBP as the substrate. Equal amounts of the immuno-precipitated (IP) MPK6 mutant proteins were used for the assays. Phosphorylation of MBP by the MPK6 mutants (top panel). Immunoblot (IB) analyses with the anti-MPK6 antibody indicated that equal amounts of the MPK6 mutants (bottom panel) were used in each reaction. (**A**) The MPK6 mutants were transiently expressed in tobacco leaves and immuno-precipitated (IP). The kinase activities of the MPK6 mutants were tested. (**B**) The MPK6 mutants were expressed in transgenic *Arabidopsis* plants and immuno-precipitated (IP). The kinase activities of the MPK6 mutants were tested.

To test the kinase activities of MPK6 and the mutants *in planta*, the *MPK6* mutant genes were first transiently transformed into tobacco leaves and HA-tagged MPK6 and the mutant proteins were then pulled down from the leaf extracts. MPK6^WT^, MPK6^K92R^, MPK6^F364L^, MPK6^F366L^, and MPK6^F368L^ were expressed well, but unfortunately, MPK6^E286S^ and MPK6^E289G^ were not expressed in tobacco leaves (Fig. 5A bottom panel) for currently unknown reasons. The in-gel kinase activity assay showed that MPK6^F364L^ and MPK6^F366L^ possessed significantly increased levels of kinase activity and MPK6^F368L^ exhibited a slightly increased level of kinase activity compared with MPK6; however, the kinase activity of MPK6^K92R^ was undetectable (Fig. 5A top panel). Then, we generated a variety of permanent transgenic *Arabidopsis* plants that stably expressed HA-tagged MPK6^WT^, MPK6^K92R^, MPK6^F364L^, MPK6^F366L^, and MPK6^F368L^. The HA-tagged proteins were immuno-precipitated from the plants and equal amounts of the MPK6^WT^ and mutant proteins were used for the kinase activity assay using the in-gel kinase activity assay. Figure 5B showed that MPK6^F364L^ and MPK6^F368L^ possessed much higher levels of kinase activity and MPK6^F366L^ exhibited a slightly higher level of kinase activity compared with MPK6 in the absence of active MKK; however, the kinase activity of MPK6^KR^ was undetectable. The results suggest that the substitution of F364, F366, and F368 in the L16 loop to Leu allowed MPK6 to obtain an intrinsic kinase activity both *in vitro* and *in planta*.

**Figure 6:**
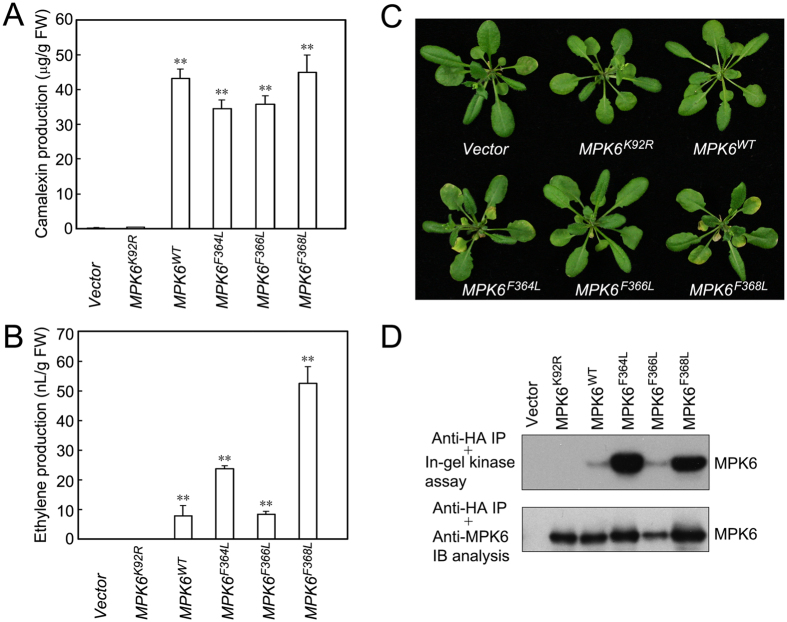
Measurements of camalexin and ethylene production and observation of leaf senescence in the MPK6 mutants transgenic *Arabidopsis* plants. The amount of camalexin (**A**) and ethylene (**B**) produced by the two-week-old MPK6 mutants transgenic seedlings were measured 12 hours after transgene induction. (**C**) Early leaf senescence of the four-week-old MPK6 mutant transgenic plants was observed 7 days after transgene induction. (**D**) MPK6 mutants kinase activities (top panel) and protein levels (bottom panel) in the transgenic *Arabidopsis* plants used for the camalexin and ethylene measurements and leaf senescence observations. The MPK6 mutant proteins were immuno-precipitated (IP) from 15 μg of total proteins and subjected to in-gel kinase assays and immunoblot analyses. In (**A**,**B**), the data represent the means ± standard deviations of three biological replicates for each treatment. Asterisks indicate statistically significant differences between the mutant seedlings and vector control seedlings. **P < 0.01 (paired *t* test).

Previous studies have reported that the activation of MPK3/MPK6 by upstream MKKs in *Arabidopsis* transgenic plants is involved in the regulation of multiple biological processes. To test whether the MPK6 active mutants created in this study could functionally mimic MKK9-activated MPK6, we detected camalexin and ethylene production^24^ and senescence^25^ in transgenic plants after induction of the MPK6 active mutant proteins. As shown in Fig. 6, the transgenic plants expressing MPK6^WT^, MPK6^F364L^, MPK6^F366L^, and MPK6^F368L^ produced comparable, high levels of camalexin; the amounts were 43.2 μg, 34.6 μg, 35.8 μg, and 44.9 μg, respectively. The plants expressing MPK6^F364L^ and MPK6^F368L^ produced approximately 3- and 6.6-folds more ethylene than did those expressing MPK6^WT^, whereas the plants expressing MPK6^F366L^ produce a similar level of ethylene as did plants expressing MPK6^WT^. The plants expressing MPK6^F364L^ and MPK6^F368L^ showed an early leaf senescence phenotype; however, the early leaf senescence phenotype was not observed in plants expressing MPK6^WT^ and MPK6^F366L^. As a kinase inactive control, the expression of MPK6^KR^ did not induce any detectable ethylene or camalexin production or the early leaf senescence phenotype in the transgenic plants. These results suggest that the active mutants MPK6^F364L^ and MPK6^F368L^ function to induce ethylene production and early leaf senescence independent of the activation of the upstream MKKs.

## Discussion

In this study, we showed that the overall structure of *Arabidopsis* MPK6 is similar to the known non-plant MAPK structures and contains all of the structural elements that are present existed in those MAPKs^3^,^4^,^5^,^32^. However, salient differences have been observed in the glycine-rich loop, L16-loop and MKI in MPK6. The results imply that MPK6 shares common structural features with other MAPKs, but also possesses specific mechanisms of activation and interactions with MAPKK, phosphatase, and substrates.

The glycine-rich loop is part of the ATP binding pocket in MAPKs and has been reported to over the nucleotide when ATP binding occurs. Compared with inactive ERK2 and p38α, the glycine-rich loop in MPK6 folds down onto the ATP binding pocket and is closer to the phosphorylation lip. This localization is somewhat similar to that in active ERK2 (as ERK2-P2) and active p38α^40^,^43^, and may facilitate the positioning of ATP and the catalytic activity of MPK6. The MKI in the known structure of non-plant MAPKs contains two helixes, α1L14 and α2L14, which have been shown to be required for the interactions between MAPKs and their regulators, activators, substrates, and lipids^44^,^45^. Mutation of specific residues in α2L14 of ERK2 impairs its interaction with upstream MAPKKs and regulators and affects its binding to nucleoporins^44^,^46^. In ERK2, Ile254 of the MKI contacts the phosphorylation lip, but, upon activation, the interaction in ERK2-P2 is lost due to the mobilization of α1L14 and α2L14 and lip refolding^3^,^40^. Studies on p38 family kinases have shown that a portion of α1L14 is responsible for kinase autophosphorylation and autoactivation^36^. The MKI in MPK6 is quite different from other non-plant MAPKs due to the absence of α1L14 in the MKI of MPK6. The existence of a cluster of acidic residues (Glu285-Glu287 and Glu289) in this region of MPK6 may be the reason for this differences. Initially, we speculated that the disruption of the acid patch in MKI might affect the kinase activity of MPK6 or its activation by MKK5; however, only the mutation of Glu289 to Gly weakly reduced MPK6 basal activity (Fig. 4). The result suggests that this acid patch may perform other unknown functions. The acid patch was also observed in several other MPKs in plants (e.g., MPK1, MPK2, MPK10, and MPK13 in *Arabidopsis* and OsMPK1, OsMPK3, OsMPK4, and OsMPK5 in rice) when analyzing the MAPK sequences from plants (here *Arabidopsis* and rice) (Figure S1). The differences imply that these MAPKs may interact with their activators, regulators, and substrates through a different mechanism.

MAPKs are a family of kinases with multiple members, that function in many signalling pathways. The dual phosphorylation of Thr and Tyr in the TXY motif of MAPKs by the upstream MAPKKs is the common mechanism for their activation, although two alternative MAPKK-independent mechanisms have been reported for p38α activation (through an interaction with TAB1 or by the phosphorylation of ZAP-70 tyrosine kinase on Tyr323 of p38 α)^47^,^48^. Because mutations of the Thr and Tyr to Glu or Asp cannot generate constitutively active MAPK, extracellular stimuli or active MAPKK are generally used to activate the MAPK to study the function of MAPK *in vivo*. Many reports have shown that a single MAPKK can usually activate more than one downstream MAPK and stimulate multiple cellular responses^10^,^18^,^19^,^49^,^50^, which makes it more difficult to determine the specific role of a given MAPK. Therefore, the generation of a constitutively active MAPK mutant and its expression in certain biological systems could be a powerful tool to study the specific functions of individual MAPKs. By introducing libraries of mutant *HOG1* genes into the *pbs2*Δ yeast strain mutant and the library of mutant *ERK2* genes into the *mkk1*Δ/*mkk2*Δ yeast strain, respectively, a series of constitutively active Hog1 and ERK2 kinase mutants were obtained^51^,^52^,^53^. Most of the mutations are in the L16 loop^52^. Based on the activating mutations in Hog1, human p38 active mutants were also generated^41^,^52^. The structural analysis of the p38 active mutants revealed that changes in the conformation of the L16 loop promoted kinase activation^42^. Recently, the yeast functional screen strategy was also used to generate plant MAPK active mutants^54^. Mutation of Tyr144 in β5 and Asp218/Glu222 in the phosphorylation lip of MPK6 and the equivalent position amino acids in MPK3 and MPK4 produced increased intrinsic kinase activity of these MAPKs *in vitro* and in yeast. Expression of the MPK4 mutants in transgenic *Arabidopsis* plants revealed that only the MPK4^D198G/E202A^ showed constitutive kinase activity *in planta*. An analysis of the MPK4^D198G/E202A^-expressing lines clarified the roles of MPK4 in regulating the defence responses and stomata opening. In this study, we generated a few mutants of MPK6 based on the MPK6 crystal structure and mutants generated in Hog1, ERK2, and p38 and detected their kinase activities. The activity assay showed that some mutations in L16 of MPK6, particularly Phe364 and Phe366 to Leu, could generate constitutively active kinase mutants both *in vitro* and *in planta*. Because alterations in the L16 conformation in MPK6 and other non-plant MAPKs (e.g., p38 and ERK2)^40^,^41^,^42^ are correlated with their kinase activities, changes in the conformation of L16 loop may be a common mechanism of MAPK activation.

In *Arabidopsis*, MKK2 activates MPK4/MPK6 to regulate cold and salt responses^18^; MKK3 activates MPK6 to participate the regulation of pathogen and JA responses^17^; MKK4/MPK5 activate MPK3/MPK6 to mediate the regulation of H_2_O_2_ production^19^, stomata and ovule developmen^20^,^55^, and defence responses^21^,^22^,^56^; MKK4/MKK5/MKK9 activate MPK3/MPK6 to promote camalexin and ethylene biosynthesis^24^,^30^, and MKK9 activates MPK3/MPK6 to regulate phosphate acquisition, leaf senescence, salt stress responses^16^,^25^. All of these findings suggest that MPK6 is a superstar MAPK; however, the activation of multiple MAPKs by the constitutively active MKKs in these experiments may lead to an over-estimation of MPK6 function. An analysis of the MKK-independent MPK6 active mutants should facilitate a better understanding of the exact roles of MPK6. In this study, we generated a series of MPK6 mutant transgenic plants and analyzed the ability of MPK6 to regulate camalexin and ethylene production and leaf senescence. The expression of MPK6^WT^, MPK6^F364L^, MPK6^F366L^, and MPK6^F368L^ could induce high but comparable levels of camalexin production, whereas only the expression of MPK6^F364L^ and MPK6^F368L^ could induce high levels of ethylene production and the leaf senescence phenotype (Fig. 6). Because MPK6^F364L^ and MPK6^F368L^ have higher kinase activities and MPK6^WT^ and MPK6^F366L^ only have a basal level of kinase activity in transgenic plants, we suggest that the activation of MPK6 should regulate ethylene production and leaf senescence. A further analysis of these mutants will help us understand the specific roles of MPK6 in regulating stress responses and developmental processes.

## Materials and Methods

### Vector Construction

DNA fragments encoding *Arabidopsis thaliana* (ecotype “Columbia”) MPK6 (At2g43790) and MPK6 truncations were PCR-amplified from the previously constructed *p*ET30a-*MPK6* vector^24^. The PCR fragments were digested with *Nde* I/*Sal* I and cloned into the modified *p*GEX-4T-2 vector. The modified *p*GEX-4T-2 vector contains a sequence encoding the GST protein followed by a tobacco etch virus protease (TEV) cleavage site (ENLYFQG).

The *MPK6* PCR fragment was also ligated into the *p*GEM-T Easy vector, and mutations were introduced using the QuickChange site-directed mutagenesis kit (Stratagene). The *Nde* I/*Sal* I fragments from the *p*GEM-T Easy vector were cloned into the *p*ET30a vector. A 6×His tag was fused to the N-terminus of MPK6 and the mutant proteins. The *MKK5* (At3g21220) cDNA was cloned and mutated as previously described. An *Nco* I/*Sal* I fragment of *Flag*-*MKK5*^*DD*^ was cloned into the *p*ET28a vector^19^.

The resulting constructs were all confirmed by sequencing and transformed into *E. coli* BL21 strain. The primers used in this study are described in supplemental Table 1.

### Expression and Purification of the Proteins Used for Crystallization

Protein expression in the cells was induced with 0.1 mM isopropyl β-D-1-thiogalactopyranoside. The cells were collected and resuspended in buffer A (100 mM Tris-HCl, pH 7.5, 150 mM NaCl, 1 mM DTT, and 1 mM PMSF) and lysed by sonication. After centrifugation at 12,000 × *g* for 30 min, the supernatant was loaded onto a column with 2 mL of Glutathione-conjugated Sepharose 4B (GE Healthcare) beads and incubated for 1 h at 4 °C. After washing with approximately 10 column volumes of buffer B (100 mM Tris-HCl, pH 7.5, and 150 mM NaCl), the GST tag was then cleaved by adding 400 μl of 1 mg/ml TEV enzyme and incubated the mixture at 4 °C overnight. Five millilitres of additional buffer B was added, and the flow through fractions were collected. The fractions were pooled and mixed with an equal volume of buffer C (100 mM Tris-HCl, pH 7.5). After centrifugation at 14,000 × *g* for 30 min, the supernatant was loaded onto a Mono Q 5/50 column. The column was eluted with a linear gradient of 0 to 1 M NaCl in buffer C. The peak fractions containing the desired proteins were pooled and concentrated. After centrifugation at 14,000 × *g* for 30 min, the supernatant was loaded onto a Superdex 200 10/300 column equilibrated with buffer D (elution buffer, 20 mM Tris-HCl, 150 mM NaCl, 1 mM DTT, and 1 mM PMSF), and the column was eluted with the same buffer. The peak fractions with the desired proteins were pooled. MPK6 and its mutant proteins appeared to be >95% pure by SDS-PAGE analysis.

### Crystallization and Structure Determination

The GST-cleaved MPK6, MPK6_Δ1–28_, and MPK6_Δ1–33_ proteins were concentrated to 10 mg/ml. The crystals were grown in drops composed of 1 μl of protein solution, 1 μl of reservoir solution containing 100 mM citrate sodium, pH 6.2, 10–12% PEG20000 (Hampton Research), using the sitting-drop vapour-diffusion method at 22 °C. After streak seeding, the shape of crystals changed from tiny needle clusters to long, large rods. Only crystals of MPK6_(Δ1–28)_ were obtained. The crystal was mounted in 50 μl of reservoir solution with 10% DMSO and air dehydrated for 5 min. The crystal was then transferred to 50 μl of reservoir solution containing 20% DMSO and air dried for 5 min. The crystals were flash-frozen in liquid nitrogen without any other cryoprotectant.

The diffraction data were collected at the Shanghai Synchrotron Radiation Facility (SSRF) at beam line BL17U1 using a CCD detector with an exposure time of 1 s per image at a crystal to detector distance of 380 mm. The data were processed using HKL2000^57^. The crystal structure was solved using the PHASER program in the CCP4 program suite. The atomic coordinates of human ERK2 (PDB code 1ERK) were used as the starting search model^3^. The model was further refined with either PHENIX^58^ or the CCP4 program^59^. The statistics on data processing and structure refinement are listed in Table 1. The structure figures were created in PyMOL (The PyMOL Molecular Graphics System, Version 1.2r3pre, Schrödinger, LLC).

### Purification of Recombinant Proteins for Use in the Kinase Activity Assay

Protein induction and cell lysis were performed as described above. MPK6 and its mutant proteins were affinity purified using a Ni^2+^-Chelating Sepharose Fast Flow column (Amersham-Pharmacia Biotech) and the Flag-MKK5^DD^ protein was affinity purified using an anti-Flag M2 affinity gel column (Sigma-Aldrich). The phosphorylation and kinase activity of MPK6 and its mutant proteins were detected using the in solution kinase assay^24^.

### Plant Materials and Treatments

The wild type and transgenic *Arabidopsis* plants and tobacco (*Nicotiana tabacum cv. Xanthi-nc*) plants were grown and treated as previously described^24^. Two-week-old seedlings grown in liquid culture medium were used for the camalexin and ethylene production assays. To observe leaf senescence, four-week-old soil-grown *Arabidopsis* plants were treated with 5 μM dexamethasone (DEX) and the photo was taken 7 days after DEX treatment.

Fully expended leaves from six-week-old tobacco plants were used for the transient transformation experiments.

### Agrobacterium-mediated Transformation

*MPK6* and its mutant genes were cloned into a modified *p*BlueScript vector with an HA-epitope tag coding sequence at the 5′-end and the Ω sequence from tobacco mosaic virus was placed before the HA-epitope tag coding sequence^19^. The coding sequences of *HA-MPK6* were inserted into the *Spe* I/*Xho* I sites of the steroid-inducible *p*TA7002 binary vector. All the resultant constructs were transformed into *Agrobacterium tumefaciens* strain, GV3101. The transient transformation experiments in tobacco were performed as previously described^19^. Transgenic *Arabidopsis* plants were generated using the flower-dipping method^60^ and selected on 0.5× MS plates with 15 mg/litre hygromycin. DEX-treated leaf tissues were used for immuno-precipitation.

### Immunoblot, Immunoprecipitation, and Kinase Activity Assays

Total protein extraction, immunoblots, and immunoprecipitations were performed as previously described^19^. An anti-HA tag antibody-conjugated agarose beads were used in the immunoprecipitation experiments. The kinase activity of the HA-tagged MPK protein was determined using the in-gel kinase assay. The anti-MPK6 antibody was used as the primary antibody in the immunoblot assay.

In-gel kinase and in solution kinase activity assays were performed as previously described^24^. Myelin basic protein (MBP) was used as the substrate.

### Measurement of Ethylene and Camalexin Production

The measurements of the amount of ethylene and camalexin produced by the transgenic seedlings were performed as previously described^24^.

### Protein Data Bank Accession Codes

The atomic coordinates and structural factors have been deposited in the Protein Data Bank, Research Collaboratory for Structural Bioinformatics, Rutgers University, New Brunswick, NJ (http://www.rcsb.org/) with accession code 5CI6.

## Additional Information

**How to cite this article**: Wang, B. *et al.* Analysis of crystal structure of *Arabidopsis* MPK6 and generation of its mutants with higher activity. *Sci. Rep.* **6**, 25646; doi: 10.1038/srep25646 (2016).

## Supplementary Material

Supplementary Information

## Figures and Tables

**Table 1:** Data collection and refinement statistics.

	MPK6
**Data collection**	
Space group	*P*3_1_2_1_
Cell dimensions	
*a*, *b*, *c* (Å)	150.51, 150.51, 85.62
α, β, γ (°)	90.00, 90.00, 120.00
Resolution (Å)	50.00–2.90 (2.95–2.90)*
*R*_merge_^a^ (%)	7.2 (68.4)
*I*/σ*I*	16.48 (2.15)
Completeness (%)	99.5 (100.00)
Redundancy	3.6 (3.7)
**Refinement**	
Resolution (Å)	42.70–3.00 (3.107–3.0)
No. reflections	22540 (2213)
*R*_work_/*R*_free_^b^ (%)	22.6/27.2 (33.1/34.7)
Number of atoms	
Protein	5447
Ligand/ion	0
Water	6
*B*-factors	
Protein	99.40
Ligand/ion	0
Water	60.20
R.m.s. deviations	
Bond lengths (Å)	0.011
Bond angles (°)	1.37

*The values in parentheses are for the highest–resolution shell.

^a^*R*_merge_ = 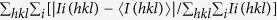, where *I*_*i*_ (*hkl*) is the intensity of reflection i and < *I* (*hkl*) > is the average of the intensities of all observations of reflection.

^b^The *R*_free_ factor was calculated for 5% of randomly chosen reflections that were not included in the refinement.
