# Supplementary material for: Analysis of crystal structure of Arabidopsis MPK6 and generation of its mutants with higher activity
Source: Sci Rep. 2016 May 10;6:25646. doi: 10.1038/srep25646 (PMC4861982; doi:10.1038/srep25646)
Supplement: Supplementary Information [file srep25646-s1.pdf]

Analysis of crystal structure of *Arabidopsis* MPK6 and generation of its mutants with higher activity

Bo Wang#, Xinghua Qin#, Juan Wu, Hongying Deng, Yuan Li, Hailian Yang, Zhongzhou Chen, Guoqin Liu, Dongtao Ren*

State Key Laboratory of Plant Physiology and Biochemistry, College of Biological Sciences, China Agricultural University, Beijing 100193, China

* Correspondence: Dongtao Ren. Email: ren@cau.edu.cn; Phone number: 86-10-62733794

# These authors are contributed equally to this work

```
AtMPK1    250 LGSQREEDLEF---------------------IDNPKAKRYIRSLPYSPGMSLSRLYPGAHVLAIDLLQKMLVFDPSKRISVSEALQHPYMAPLYDPNANPPAQVPID-LDVDED- 342
AtMPK2    250 LGSQREEDLEF---------------------IDNPKAKRYIESLPYSPGISFSRLYPGANVLAIDLLQKMLVLDPSKRISVTEALQHPYMAPLYDPSANPPAQVPID-LDVDEDE 343
AtMPK3    255 LGTPTESDLGF---------------------THNEDAKRYIRQLPNFPRQPLAKLFSHVNPMAIDLVDRMLTFDPNRRITVEQALNHQYLAKLHDPNDEPICQKPFS-FEFEQQP 348
AtMPK4    260 IGSPDDSSLGF---------------------LRSDNARRYVRQLPQYPRQNFAARFPNMSAGAVDLLEKMLVFDPSRRITVDEALCHPYLAPLHDINEEPVCVRPFN-FDFEQPT 353
AtMPK5    260 IGSPDGASLEF---------------------LRSANARKYVKELPKFPRQNFSARFPSMNSTAIDLLEKMLVFDPVKRITVEEALCYPYLSALHDLNDEPVCSNHFS-FHFEDPS 353
AtMPK6    280 IGTPSEEELEF---------------------LN-ENAKRYIRQLPPYPRQSITDKFPTVHPLAIDLIEKMLTFDPRRRITVLDALAHPYLNSLHDISDEPECTIPFN-FDFENHA 372
AtMPK7    250 VGSQQESDIRF---------------------IDNPKARRFIKSLPYSRGTHLSNLYPQANPLAIDLLQRMLVFDPTKRISVTDALLHPYMAGLFDPGSNPPAHVPIS-LDIDEN- 342
AtMPK8    326 LGTPPPESISR---------------------IRNEKARRYLSSMRKKQPVPFSHKFPKADPLALRLLERLLAFDPKDRASAEDALADPYFSGLSNSEREPTT-QPISKLEFDFER 419
AtMPK9    245 LGTPPPEAIAR---------------------IRNEKARRYLGNMRRKPPVPFTHKFPHVDPLALRLLHRLLAFDPKDRPSAEEALADPYFYGLANVDREPST-QPIPKLEFEFER 338
AtMPK10   277 IGTPSEEELGS---------------------LS-EYAKRYIRQLPTLPRQSFTEKFPNVPPLAIDLVEKMLTFDPKQRISVKEALAHPYLSSFHDITDEPECSEPFN-FDLDEHP 369
AtMPK11   257 IGSPDDSSLGF---------------------LRSDNARRYVRQLPQYPRQNFAARFPNMSVNAVDLLQKMLVFDPNRRITVDEALCHPYLAPLHEYNEEPVCVRPFH-FDFEQPS 350
AtMPK12   258 VGSPDNSSLGF---------------------LRSDNARRYVRQLPRYPKQQFAARFPKMPTTAIDLLERMLVFDPNRRISVDEALGHAYLSPHHDVAKEPVCSTPFS-FDFEHPS 351
AtMPK13   250 LGSPDDSDLDF---------------------LRSDNARKYVKQLPHVQKQSFREKFPNISPMALDLAEKMLVFDPSKRITVDEALKQPYLASLHEINEEPTCPTPFS-FDFEETA 343
AtMPK14   247 VGSQQDWDLQF---------------------IDNQKARRFIKSLPFSKGTHFSHIYPHANPLAIDLLQRMLVFDPTKRISVSDALLHPYMEGLLEPECNPSENVPVSSLEIDEN- 340
AtMPK15   312 LGTPPPEAISK---------------------IRNDKARRYLGNMRKKQPVPFSKKFPKADPSALRLLERLIAFDPKDRPSAEEALADPYFNGLSSKVREPST-QPISKLEFEFER 405
AtMPK16   247 LGTPSAEAIGR---------------------VRNEKARRYLSSMRKKKPIPFSHKFPHTDPLALRLLEKMLSFEPKDRPTAEEALADVYFKGLAKVEREPSA-QPVTKLEFEFER 340
AtMPK17   238 LGTPSPITLSR---------------------IRNEKARKYLGNMRRKDPVPFTHKFPNIDPVALKLLQRLIAFDPKDRPSAEEALADPYFQGLANVDYEPSR-QPISKLEFEFER 331
AtMPK18   247 LGTPKSETISG---------------------VRNDKARKYLTEMRKKNPVTFSQKFSKADPLALRLLQRLLAFDPKDRPTPAEALADPYFKGLSKIEREPSS-QQISKMEFEFER 340
AtMPK19   247 LGTPKSETIAG---------------------VRNEKARKYLNEMRKKNLVPFSQKFPNADPLALRLLQRLLAFDPKDRPTAAEALADPYFKCLAKVEREPSC-QPISKMEFEFER 340
AtMPK20   247 LGTPSLDTISR---------------------VRNEKARRYLTSMRKKPPIPFAQKFPNADPLSLKLLERLLAFDPKDRPTAEEALADPYFKGLAKVEREPSC-QPITKMEFEFER 340
OsMPK1    284 IGTPNEADLDF---------------------VN-ENARRYIRQLPRHARQSFPEKFPHVHPLAIDLVEKMLTFDPRQRITVEGALAHPYLASLHDISDEPVCSSPFS-FDFEQHA 376
OsMPK2    275 IGSPDDSSLGF---------------------LRSDNARRYVRSLPQYPKQQFRARFPTMSSGAMDLLERMLVFDPSKRITVDEALCHPYLASLHEIYDEPVCPAPFS-FDFEQPS 369
OsMPK3    250 LGTMSESDLEF---------------------IDNPKARRYIKSLPYTPGVPLASMYPHAHPLAIDLLQKMLIFDPTKRISVTEALEHPYMSPLYDPSANPPAQVPID-LDIDEN- 342
OsMPK4    250 LGTMSEADIEF---------------------IDNPKARKYIKTLPYTPGIPLTSMYPQAHPLAIDLLQKMLVFDPSKRISVTEALEHPYMSPLYDPSANPPVQVPID-LDIDEN- 342
OsMPK5    253 IGTPTDDELGF---------------------IRNEDARKYMRHLPQYPRRTFASMFPRVQPAALDLIERMLTFNPLQRITVEEALDHPYLERLHDIADEPICLEPFS-FDFEQKA 346
OsMPK6    270 IGSPDDSSLGF---------------------LRSDNARRYMKQLPQYPRQDFRLRFRNMSAGAVDLLEKMLVFDPSRRITVDEALHHPYLASLHDINEEPTCPAPFS-FDFEQPS 363
OsMPK7    258 LGTPSMDAISR---------------------IRNDKARRYLSSMRRKQPVPFSEKFPNVDPLALKLLQRLLAFDPKDRPTAEEALADPYFKGLAKVEREPSC-QPISKMEFEFER 351
OsMPK8    235 LGTPSMDTVTR---------------------IRNEKARRYLSSMRKKQPVPFSERFPKADPAALKLLQRLLAFDPKDRPTAEEALADPYFKGLAKAEREPSC-QPITKMEFEFER 328
OsMPK9    329 LGTPSMDTISRVLLGGDFSDPQHLSYCSLPFQVRNEKARRYLSSMRKKDPVPFSQKFPNADPLALKLLQRLLAFDPKDRPTAEEALTDPYFKGLSKIDREPSC-QPIRKLEFEFEQ 443
OsMPK10   290 LGTPSMDTISR---------------------VRNDKARRYLSSMRKKEPILFSQKFPSADPLALDLLQKLLAFDPKDRPTAEEALAHPYFKGLAKVEREPSC-QPITKMEFEFER 383
OsMPK11   248 LGTPSLDAISQ---------------------VRNDKARKYLTCMRKKQPASFSHKFLKADPLALQLLRKLLAFDPKDRPSAQEALADPYFNGLAKVEREPSC-QPIPKMEFEFER 341
OsMPK12   235 LGTPSSETLSR---------------------IRNEKARRYLSTMRKKHAVPFSQKFRNTDPLALRLLERLLAFDPKDRPSAEEALADPYFASLANVEREPSR-HPISKLEFEFER 328
OsMPK13   235 LGTPSSETLSR---------------------IRNENARGYLTGMQRKHPIPFSHKFHNADPLALRLLERLLAFDPKDRPTAEEALADPYFRGISKLSREPSR-LPVSKFEFEFER 328
OsMPK14   235 LGTPSPETISR---------------------IRNEKARRYLNSMRRKKPIPFTQKFPNADPLAMRLLERMLAFDPKDRPSAEEALADPYFKNIANVDREPSA-QPITKLEFEFER 328
OsMPK15   235 LGTPSTEAISR---------------------IRNEKARRYLSSMRRKKPIPFTQKFPNADPLALRLLERMLSFEPKDRPNAEEALADPYFRNIANVDREPSA-QPVTKLEFEFER 328
OsMPK16   333 LGSPSGETISR---------------------IRNEKARRYLGNMRKKPRVPFSQKFPGADPMALHLLERLLAFDPKDRPTAAEALTDPYFTGLANSEREPIA-QPISKLEFEFER 426
OsMPK17   327 LGTPSAESLAK---------------------IRNEKARRYLSNMRKKPRVPFTKKFPGVDPMALHLLERLLAFDPKDRPSAEEALTDPYFNGLANSEREPIA-QPISKLEFEFEK 420
```

Figure S1. Mutiple sequence alignment of *Arabidopsis* and rice MPKs.

Sequence alignment of Arabidopsis and rice MPKs were generated by ClustalW. The sequences that included the putitive α1L14 and L16 regions were presented. Amino acids were numbered. The red boxes indicated the amino acid residues which might interfere with a1L14 formation. The green boxes indicated the residues which are similar to those of the mutated residues in L16 of *Arabidopsis* MPK6 in this study. The sequences used are: AtMPK1 (At1g10210), AtMPK2 (At 1g59580), AtMPK3 (At 3g45640), AtMPK4 (At 4g01370), AtMPK5 (At 4g11330), AtMPK6 (At 2g43790), AtMPK7 (At At2g18170), AtMPK8 (At 1g18150), AtMPK9 (At 3g18040), AtMPK10 (At 3g59790), AtMPK11 (At At1g01560), AtMPK12 (At 2g46070), AtMPK13 (At 1g07880), AtMPK14 (At 4g36450), AtMPK15 (At 1g73670), AtMPK16 (At 5g19010), AtMPK17 (At 2g01450), AtMPK18 (At At1g53510), AtMPK19 (At 3g14720), AtMPK20 (At 2g42880), OsMPK1 (Os06g0154500), OsMPK2 (Os08g0157000), OsMPK3 (Os02g0148100), OsMPK4 (Os06g0699400), OsMPK5 (Os03g0285800), OsMPK6 (NM_197522), OsMPK7 (AK099472), OsMPK8 (Os01g0665200), OsMPK9 (AAT44204), OsMPK10 (NM_192924), OsMPK11 (Os06g0367900), OsMPK12 (Os06g0708000), OsMPK13 (Os02g0135200), OsMPK14 (AAS98446), OsMPK15 (Os11g0271100), OsMPK16 (NM_192298), OsMPK17 (Os05g0576800).

Table S1. Oligonucleotides used in this study

Oligo name	Oligonucleotide (5’-3’)
	Primers used for MPK6 crystalization
MPK6$_{\Delta(1\text{-}28)}$ F	GGAATTCCATATGCAGATGCCTGGGATTGAG
MPK6$_{\Delta(1\text{-}28)}$ B1	GTTAGTCGACTTATTGCTGATATTCTGGATTGA
	Primers for the truncated MPK6 proteins generation
MPK6 F1	GGAATTCCATATGGACGGTGGTTCAGGTCAACC
MPK6 B1	ACGCGTCGACTTGCTGATATTCTGGATTGAAAGC
MPK6$_{\Delta(1\text{-}28)}$ F	GGAATTCCATATGCAGATGCCTGGGATTGAG
MPK6$_{\Delta(1\text{-}33)}$ F	GGAATTCCATATGGAGAATATTCCGGCGAC
MPK6$_{\Delta(1\text{-}56)}$ F	GGAATTCCATATGGAGGTCACCGCTAAG
MPK6$_{\Delta(1\text{-}70)}$ F	GGAATTCCATATGAAGGGTGCTTATGGC
	Primers used for the MPK6 activity mutants generation
MPK6 F2	CATATGGACGGTGGTTCAGGT
MPK6 B2	CTATTGCTGATATTCTGGATTGAAA
MPK6^{K92R} F	AGAGCGTTGCGATTAGGAAAATTGCTAACG
MPK6^{K92R} B	CGTTAGCAATTTTCCTAATCGCAACGCTCT
MPK6^{F364L} B	TTCAAAATCAAAGTTGAGAGGTATTGTACACT
MPK6^{F366L} F	ACAATACCTTTCAACCTTGATTTTGAAAACCA
MPK6^{F366L} B	CATGGTTTTCAAAATCAAGGTTGAAAGGTATT
MPK6^{F368L} F	CCTTTCAACTTTGATCTTGAAAACCATGCACT
MPK6^{F368L} B	AGAGTGCATGGTTTTCAAGATCAAAGTTGAA
MPK6^{E286S} F	CATCAGAATCAGAGCTTGAGTTCTTGAAC
MPK6^{E286S} B	GTTCAAGAACTCAAGCTCTGATTCTGATG
MPK6^{E289G} F	CATCAGAAGAAGAGCTTGGGTTCTTGAAC
MPK6^{E289G} B	GTTCAAGAACCCAAGCTCTTCTTCTGATG
MPK6$^{E286G/E286S}$ F	CATCAGAATCAGAGCTTGGGTTCTTGAAC
MPK6$^{E286G/E286S}$ B	GTTCAAGAACCCAAGCTCTGATTCTGATG
